# LocoGSE, a sequence-based genome size estimator for plants

**DOI:** 10.3389/fpls.2024.1328966

**Published:** 2024-03-14

**Authors:** Pierre Guenzi-Tiberi, Benjamin Istace, Inger Greve Alsos, Eric Coissac, Sébastien Lavergne, Jean-Marc Aury, France Denoeud

**Affiliations:** ^1^ Génomique Métabolique, Genoscope, Institut François Jacob, CEA, CNRS, Univ Evry, Université Paris-Saclay, Evry, France; ^2^ The Arctic University Museum of Norway, UiT The Arctic University of Norway, Tromsø, Norway; ^3^ Univ. Grenoble Alpes, Univ. Savoie Mont Blanc, CNRS, LECA (Laboratoire d’Ecologie Alpine), Grenoble, France

**Keywords:** genome size estimation, genome size, ploidy, genome-skimming, environmental DNA, plant genomics, 1C, 1Cx

## Abstract

Extensive research has focused on exploring the range of genome sizes in eukaryotes, with a particular emphasis on land plants, where significant variability has been observed. Accurate estimation of genome size is essential for various research purposes, but existing sequence-based methods have limitations, particularly for low-coverage datasets. In this study, we introduce LocoGSE, a novel genome size estimator designed specifically for low-coverage datasets generated by genome skimming approaches. LocoGSE relies on mapping the reads on single copy consensus proteins without the need for a reference genome assembly. We calibrated LocoGSE using 430 low-coverage Angiosperm genome skimming datasets and compared its performance against other estimators. Our results demonstrate that LocoGSE accurately predicts monoploid genome size even at very low depth of coverage (<1X) and on highly heterozygous samples. Additionally, LocoGSE provides stable estimates across individuals with varying ploidy levels. LocoGSE fills a gap in sequence-based plant genome size estimation by offering a user-friendly and reliable tool that does not rely on high coverage or reference assemblies. We anticipate that LocoGSE will facilitate plant genome size analysis and contribute to evolutionary and ecological studies in the field. Furthermore, at the cost of an initial calibration, LocoGSE can be used in other lineages.

## 1 Introduction

Genome size is a trait that has been shown to vary greatly between eukaryotes, but does not correlate with organismal complexity (Mirsky and Ris, 1951; Cavalier-Smith, 1978; Gregory, 2005). Notably, in land plants, there is a, 2400-fold variation of genome size between different species (Pellicer et al., 2018), which has been shown to be caused by lineage-specific insertion/excision dynamics of DNA elements such as retrotransposons (Bennetzen et al., 2005; Grover et al., 2008; Pellicer et al., 2018; Chase et al., 2023). For example, genome size increases have been related to retrotransposon invasions in Poaceae (Sanmiguel and Bennetzen, 1998; Hawkins et al., 2006; Piegu et al., 2006; Dai et al., 2022), Melanthiaceae (Pellicer and Leitch, 2014; Pellicer et al., 2021) or Gymnosperms (Morse et al., 2009; Ohri, 2021). In addition to retrotransposon invasions, giant genomes are thought to have arisen because of the lack of DNA removal (Kelly et al., 2015). Besides, whole genome duplications are frequent in plants (Jaillon et al., 2007; Jiao et al., 2012, Jiao et al., 2014; Murat et al., 2017; Ren et al., 2018) and contribute to a lesser extent to genome size variations (Pellicer et al., 2018). They also result in variable ploidy levels between plant species as well as inside populations (Weiss-Schneeweiss and Schneeweiss, 2013).

Interest in plant genome size is high not only because of the need to estimate sequencing efforts required to obtain full genome sequences (Kelly et al., 2012; Li and Harkess, 2018; Pellicer and Leitch, 2020) but more importantly because this trait has been shown to be of evolutionary and ecological significance (Greilhuber and Leitch, 2013; Pellicer et al., 2018; Blommaert, 2020). The Kew Plant DNA C-values Database (Pellicer and Leitch, 2020); https://cvalues.science.kew.org/) is a valuable resource for plant genome sizes and provides C-values (i.e. total amount of DNA in the unreplicated haploid nucleus, or holoploid genome size (Greilhuber et al., 2005) for more than 12,000 plant species. These measures are generally obtained by flow cytometry (Dolezel et al., 2007; Sliwinska et al., 2022; Temsch et al., 2022), an experimental technique that usually requires live or frozen tissues with intact cells. Such requirements are not always easy to fulfill, for instance for botanists who work with (sometimes ancient) herbarium collections. Sequencing data provide an interesting alternative to estimate genome size (Pflug et al., 2020). Indeed, genome skimming approaches aimed at obtaining plant phylogenetic barcodes have been expanding over the last decade (Coissac et al., 2016; Li et al., 2019; Nevill et al., 2020; Fu et al., 2022). These approaches rely on low coverage short-read sequencing (usually less than 10 Million Illumina read pairs) in order to assemble chloroplastic genomes (or targeted barcode genes) and were shown to be applicable even on ancient herbarium samples (Alsos et al., 2020). Here, we present LocoGSE, a software to estimate monoploid genome size “1Cx” from such very low coverage datasets.

The monoploid genome size (1Cx) is the DNA content of the whole chromosome complement, irrespectively of the degree of polyploidy (Greilhuber et al., 2005). For diploid species, 1Cx is equal to 1C. Usually, genome size estimators do not specify which type of “genome size” they are estimating. In reality, all sequence-based genome size estimators are estimating monoploid (1Cx) rather than holoploid (1C) genome size. Such a distinction might not be essential for lineages that are mostly diploid [for instance insects (Pflug et al., 2020)], but when one wants to analyze plant genomes, where polyploidization events are frequent, it is important to have a clear definition of the genome size that is being estimated.

Previously described sequence-based methods for genome size estimation belong to two main categories: k-mer-based or mapping-based approaches (Sun et al., 2018; Liu et al., 2020; Pflug et al., 2020). K-mer-based approaches only require raw sequences but at a relatively high depth of coverage, usually above 30X for the most commonly used software GenomeScope (Vurture et al., 2017; Ranallo-Benavidez et al., 2020). These methods can not distinguish between two (or more) subgenomes with low degrees of heterozygosity, and will thus always predict the 1Cx genome size rather than 1C. In fact, GenomeScope 2.0 uses a ploidy level as input (default=2) and predicts a “genome haploid length” that actually corresponds to 1Cx and should be multiplied by the ploidy to obtain the 2C value (DNA content of a diploid cell). Hozza et al. designed a k-mer based approach (CovEst) for lower depths. Their tests showed promising results at depths of coverage as low as 1X but they were performed only on simulated genomes and a small bacterial (*E. coli*) genome (Hozza et al., 2015). The performance and optimal depth threshold for CovEst still need to be estimated on large and complex genomes. Pflug et al. showed its efficiency on various insects and three model organism species (*Arabidopsis thaliana*, *Caenorhabditis elegans*, and *Drosophila melanogaster*), but the depths of coverage in their datasets were all over 30X (Pflug et al., 2020). Recently another package, RESPECT, was developed specifically for low-coverage genome skims. It was shown to perform better than CovEst to predict genome size from low coverage datasets, even at very low depth of coverage (0.5X) (Sarmashghi et al., 2021). However, as specified by the authors, RESPECT is designed and optimized to work with low coverage data, and should not be used with sequencing depths above 5X. That causes a problem when one wants to estimate the size of a genome without prior knowledge, since the genome size needs to be known in order to calculate the number of reads to use as input to the program. Finally, one needs to keep in mind that k-mer based methods are very sensitive to heterozygosity (Pucker, 2019) and thus need to be used cautiously. Current mapping-based approaches need to map the reads onto an assembly [ModEst (Pfenninger et al., 2022)] and some also necessitate a reference single copy gene set, like MGSE (Pucker, 2019) and Gnodes (Gilbert, 2022) for short reads, and Depthsizer (Chen et al., 2022) for long reads. These approaches imply that assembly and sometimes also annotation have been performed on the genome studied, which requires high sequencing depth. Consequently, they are not suited for very low coverage datasets, such as the ones produced by genome skimming projects.

Our genome size estimator, called LocoGSE (Low coverage based Genome Size Estimator), is a mapping-based approach that does not rely on a genome assembly, since the reads are mapped on a reference dataset of single copy genes (protein consensus) instead. Thus, it is particularly suitable for very low coverage short reads datasets. We calibrated LocoGSE using 430 Angiosperm low-coverage genome skimming datasets. Then, we performed a benchmark to compare its results and performances with other available genome size estimators, on plant datasets with various sequencing depths and properties (heterozygosity, ploidy). We show that monoploid genome size estimations made by LocoGSE are accurate even at very low coverage (<1X) and on highly heterozygous samples. Interestingly, the genome size predictions remain accurate at higher coverage, which allows its use without any prior knowledge about the size of the genome analyzed. Monoploid genome size estimations are also stable across individuals with varying ploidy levels.

## 2 Methods

### 2.1 Rationale

**Figure 1:**
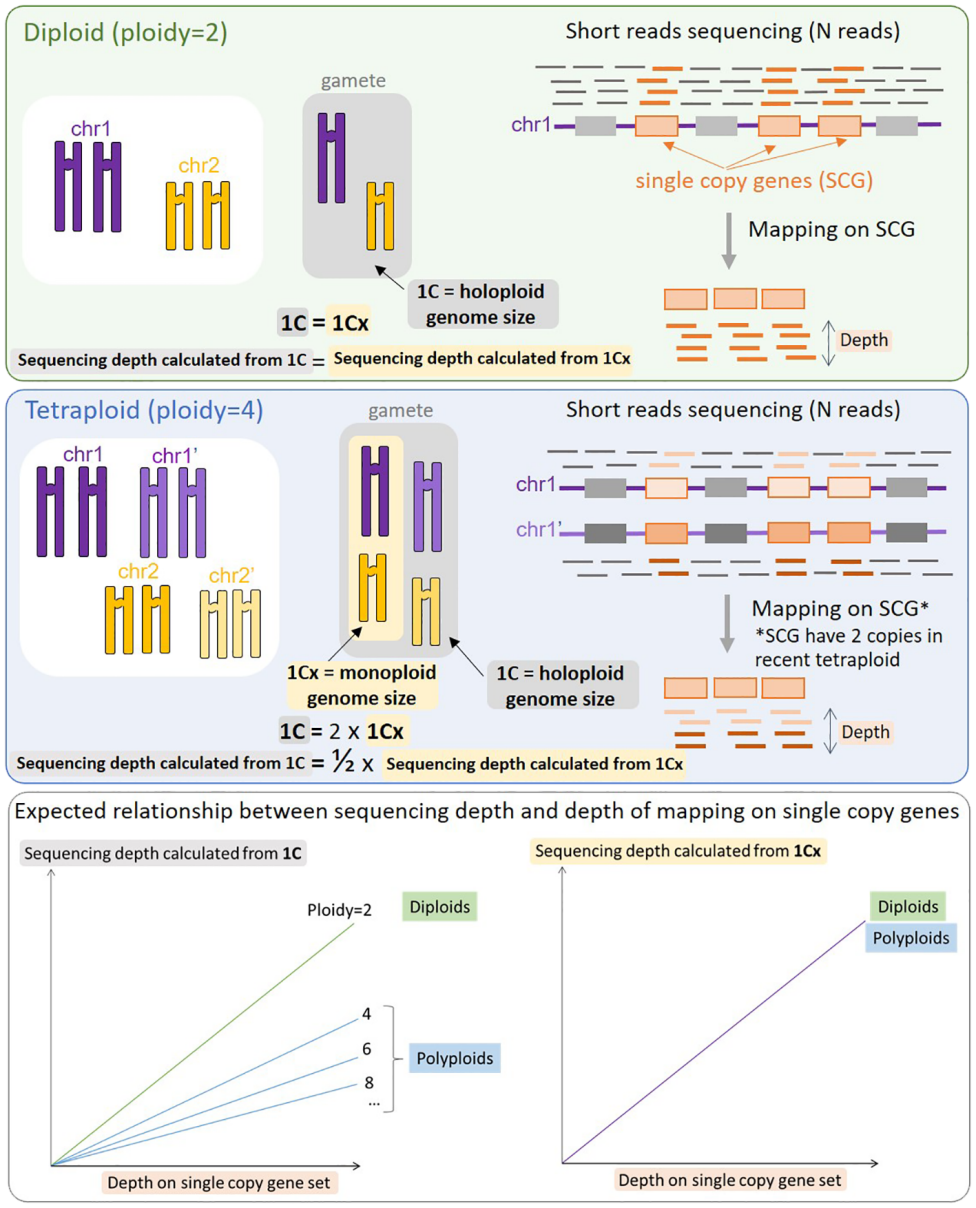
Schematic representation of the process of mapping reads on single copy genes (SCG) and expected results for a diploid species (top panel) and a tetraploid species (middle panel). When the same number of reads (N) is mapped, the resulting mapping depth on SCGs will be identical for a diploid and a recent tetraploid, provided that all SCGs are still in the duplicated state. The resulting relationship between sequencing depth and depth of mapping on SCG is displayed on the bottom panel: the slope is the same between the diploid and the tetraploid (and any other level of ploidy) when considering 1Cx (right), but decreases with the level of ploidy when considering 1C (left). With no prior knowledge of the ploidy level of the organism sequenced, mapping on SCG genes provides a stable estimate of 1Cx.

All sequence-based genome size predictors rely on the Lander-Waterman equation (Lander and Waterman, 1988), C = L/G, where G corresponds to genome size, L corresponds to cumulative length of sequenced nucleotides, C corresponds to sequencing depth of coverage. The aim is thus to estimate C in order to calculate G. The assumption behind LocoGSE and other mapping-based methods targeted on single-copy sequences (Pucker, 2019; Chen et al., 2022) is that the average depth on a set of single-copy sequences (usually single copy genes) is representative of the depth of coverage on the entire genome. Estimating the depth on a set of single-copy sequences should then allow us to estimate C and then G. However, it is important to take into account possible whole genome duplications (WGD) that may lead to various degrees of ploidy. If a tetraploidy event occurred recently, all the genome is duplicated, leading to a double genome size, but a similar proportion of single copy genes relative to the rest of the genome. At a given sequencing depth, the depth on single copy genes will then be the same for a recent tetraploid as for a diploid, and reflect the size of the monoploid genome, 1Cx (Greilhuber et al., 2005) (**Figure 1**). Consequently, genome size estimators based on mapping on single copy genes will always predict monoploid genome size (1Cx) rather than holoploid genome size 1C, as do k-mer based estimators. Conversely, the experimental protocol aiming at measuring the DNA content in cells, flow cytometry, is obviously measuring 1C genome sizes (Pellicer and Leitch, 2014). Thus, 1Cx genome size estimators have a promising application: they can be used to complement flow cytometry analysis, by estimating the ploidy level of plant specimens (by dividing 1C measurements by 1Cx estimations).

Importantly, LocoGSE is a mapping-based estimator that does not rely on a genome assembly. Rather than mapping the reads on a genomic sequence, LocoGSE maps the reads on protein sequences. Short reads are translated into amino-acid sequences and aligned on protein sequences derived from consensus sequences of single copy genes that are shared across all plant lineages.

### 2.2 Implementation

LocoGSE is coded in Python, runs on a Linux operating system and is included into a Conda environment, since it contains several calls to external programs. It is freely available at https://github.com/institut-de-genomique/LocoGSE.

The program comprises four steps (**Supplementary Figure S1**). First, sequencing reads are trimmed to 100 nucleotides with Cutadapt (v3.5) (Martin, 2011). In case inputted reads are 100nt long, the users can use the “–no-trim” option to skip the trimming step. Otherwise, the trimming to 100 nt should always be performed: this step is important since the calibration step was performed on 100 nt reads and mapping efficiency is highly dependent on the length of the reads as longer reads are more prone to overlap exon/intron junctions. In a second step, reads are aligned on a set of single copy proteins (by default OneKP ancestral proteins (see section 2.3.3) but the option –busco allows to use BUSCO Embryophyta instead: both protein datasets are provided with the program). The alignment is performed with DIAMOND (Buchfink et al., 2021) (v2.0.14, command “diamond blastx”, with e-value parameter set to 0.00001). One best hit per read is then selected and the depth of mapping on each protein is calculated. Subsequently, a filtering step is performed to remove outlier proteins, too highly or too poorly covered compared to the whole single copy protein gene set. Outlier proteins are determined by computing the mapping depth per protein then calculating the Z-Score for each protein and removing the ones with Z-score > 1.96 or< -1.96 (threshold corresponding to P<0.05). These could correspond to genes that have been lost or duplicated in the considered species, or proteins harboring unspecific domains. Finally, the genome size estimation is performed using the following equation, modified from Lander-Waterman (Lander and Waterman, 1988):


$$\text{G }=\;\frac{L}{\text{β x SCP depth}}$$

where L is the cumulative length of the reads used as input for mapping, β is the regression coefficient calculated during the calibration step (default is 1), and SCP depth is the overall depth of mapping of reads on single copy proteins (SCP) after removing the outliers (i.e. total length of reads mapped on all retained SCP divided by the cumulative length of all retained SCP).

A calibration step was performed for Angiosperm lineages in order to calculate β coefficients (see section 2.3). In consequence, for Angiosperm genome size prediction, the user can either provide a plant lineage (listed with the –listlineages option) or a plant family (listed with the –listfamilies option) (**Supplementary Figure S2A**).

Alternatively, the user can apply LocoGSE to other lineages (animals for instance), and other single copy proteins (BUSCO for instance) and perform their own calibration as explained in the dedicated wiki page: https://github.com/institut-de-genomique/LocoGSE/wiki/2.Linear-regression before providing a slope value with the –slope option (**Supplementary Figure S2B**).

### 2.3 Calibration on Angiosperms

Since the genomic reads are mapped on protein sequences (after 6 frame translation), we do not expect to obtain the real sequencing depth when calculating the depth on the single copy protein set. Indeed, reads corresponding to the targeted loci will not be mapped when they happen to fall onto exon/intron or CDS/UTR junctions. Therefore, the method needs to be calibrated using a set of known 1Cx values. The outcome of the calibration is expected to be impacted by the structure of the genes and in particular by the average number of coding exons in the group of species studied.

#### 2.3.1 Retrieving 1C values from Kew db and calculating 1Cx reference values

We extracted prime 1C estimates and associated ploidy levels from Kew Plant DNA C-values database (https://cvalues.science.kew.org/). Estimates with no documented ploidy level in Kew db were not considered. We calculated 1Cx with the following formula:


$$1Cx=2*\;\left(\frac{1C}{ploidy}\right)\;\left(\mathrm{by}\;\mathrm{definition},\;1\text{C}\;=\;1\mathrm{Cx}\;\mathrm{when}\;\mathrm{ploidy}=2\right)$$

When prime estimates for several cytotypes (specimens with different ploidy levels) were available, we discarded species for which there was more than 10% variation among cytotypes in the calculated 1Cx. Among those is the notable example of *Prospero autumnale* for which cytotypes with various chromosome numbers (5, 6, or 7 pairs) and genome sizes have been described (Vestek et al., 2019). For the remaining species, we calculated the mean 1Cx value between cytotypes and considered it as the reference 1Cx for the species.

#### 2.3.2 Angiosperm readsets

We used 430 Angiosperm genome skimming read sets (2 x 100 paired ends illumina reads) from arctic and alpine sampling campaigns (Olofsson et al., 2019; Alsos et al., 2020; Pouchon et al., 2022; Smyčka et al., 2022), for which reference 1Cx genome sizes could be calculated from Kew Plant DNA C-values database (**Supplementary Table S1**). The samples are broadly distributed among the Monocot and Dicot lineages and their phylogenetic distribution is comparable to that of the species in OneKP (**Supplementary Figure S3**). Magnolids are absent at the moment, but new calibrations will be performed once more read sets are made public from the PhyloAlps campaign, and include also Gymnosperms, and basal Streptophytes. The sequencing depth of coverage of the 430 read sets varies from 0.017X to 10.13X, with 55% of the samples with depth<1, and 29% between 1 and 2 (**Supplementary Figure S4**).

#### 2.3.3 Selection of the single copy protein set

We compared two plant single copy gene sets to use for plant genome size estimation in LocoGSE: the widely used BUSCO Embryophyta ancestral proteins (Simão et al., 2015; Manni et al., 2021) (https://busco-data.ezlab.org/v4/data/lineages/embryophyta_odb10.2019-11-20.tar.gz), and the OneKP proteins (Leebens-Mack et al., 2019). OneKP multiprotein alignments were downloaded at https://github.com/smirarab/1kp/blob/master/alignments/alignments-FAA.tar.bz. Consensus sequences were created from multiple alignments using the HMMER3 package version 3.1b1 (Mistry et al., 2013) with the hmmemit function and default parameters.

OneKP contains 1,178 plant transcriptomes (including 658 Monocot and Dicot) that are more diverse phylogenetically than species represented in BUSCO Embryophyta (that contains only 90 Monocot and Dicot genomes, almost half of which are rosids) (**Supplementary Figure S3A**). Consequently, the consensus derived from OneKP is more representative (in terms of %identity of the matches) of all Angiosperm lineages than BUSCO (**Supplementary Figure S3B**). Moreover, OneKP contains only 410 ancestral protein sequences, less than half of BUSCO Embryophyta (ODB10) that contains 956 sequences: mapping the reads on OneKP should thus be faster.

Finally, the correlation between the theoretical depth and depths obtained from mapping reads on single copy consensus proteins are slightly better when using OneKP compared to BUSCO (**Supplementary Table S2**). For all these reasons, we chose to use the OneKP consensus proteins as the default protein database when predicting plant genome sizes with LocoGSE. The databases (OneKP protein consensus sequences and BUSCO Embryophyta) are provided with the program.

#### 2.3.4 Linear regression between depth on OneKP and sequencing depth of coverage

We observe that the depth of mapping on the OneKP single copy gene set (calculated using LocoGSE, **Supplementary Figure S2B**) and the theoretical sequencing depth calculated from Kew 1Cx are very well correlated (Pearson correlation= 0.89, Pval=2.2e-16, **Supplementary Table S2**). In addition, their relationship is close to linear (**Supplementary Figure S5A**). We noticed that P-values for Pearson correlations and R2 of the regressions were slightly higher when separating plant lineages (**Supplementary Table S2**) rather than considering all samples together. Thus, we performed separate calibrations for each lineage. Our approach uses a linear regression to calculate the coefficient (β) by which to multiply the depth on OneKP single copy proteins in order to obtain the sequencing depth with the following formula:


Estimated 1Cx depth= β x OneKP depth

**Figure 2:**
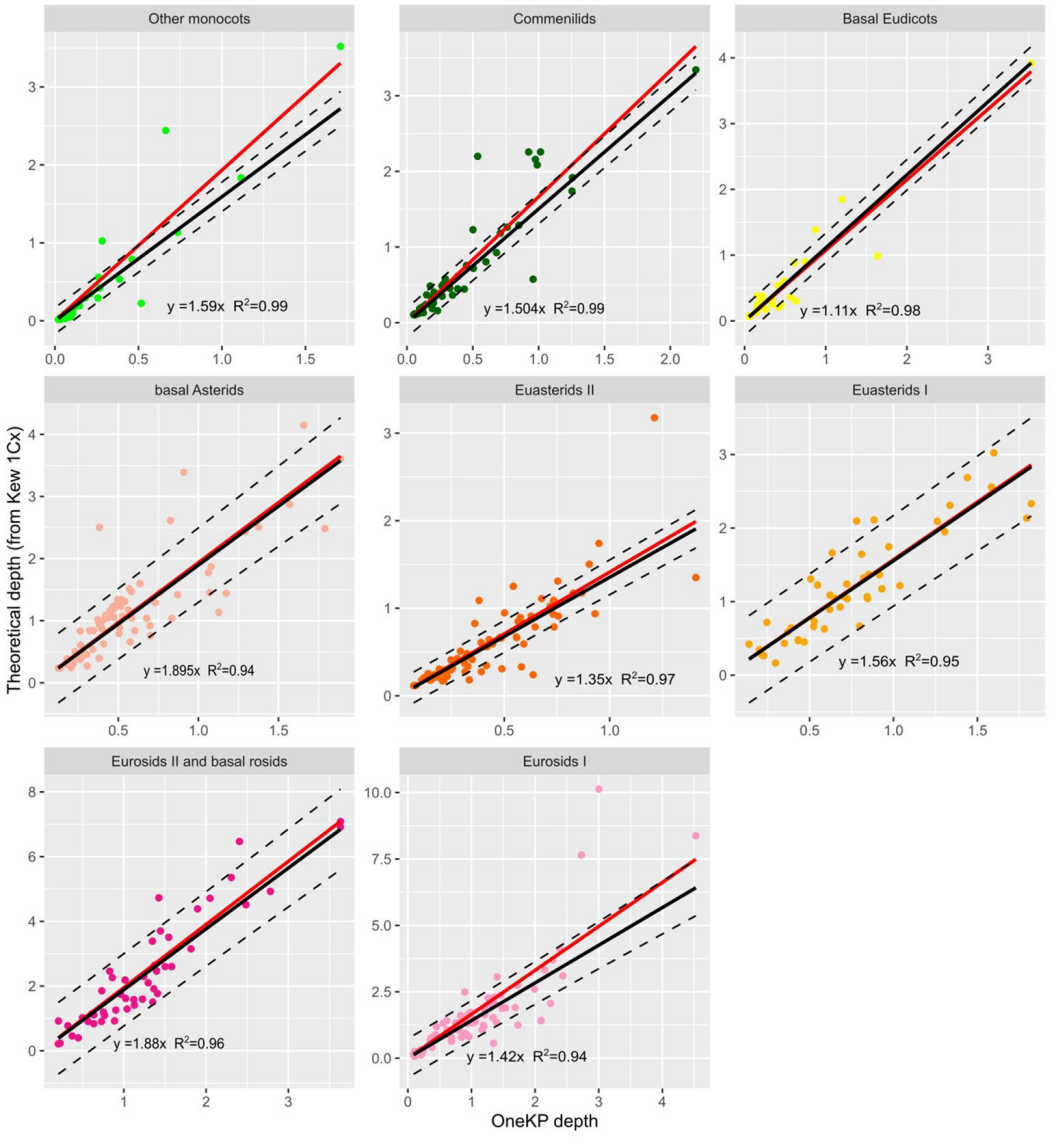
Relationship between depth on OneKP single copy genes and theoretical depth (calculated from Kew 1Cx) in the training set, for 8 plant lineages. Black line is the regression line obtained after robust regression, red line is the regression line obtained with standard regression. Regression line equations and R coefficients are displayed for each lineage.

For each group, we estimated the β coefficient with a robust linear regression (**Figure 2**). Robust linear regressions were performed with the Rpackage *robust* (*lmrob* function) and allowed to minimize the influence of outliers on the results of the regression. The β coefficients vary from 1.11 to 1.895 across lineages, and the coefficient obtained on all lineages together is of 1.56 (**Supplementary Table S2**), and could be used in the absence of information on the plant lineage.

In order to check the rationale of our approach, we also calculated the Pearson correlation between the depth on OneKP and the sequencing depth calculated from Kew 1C: as expected, the correlation coefficient (0.83) is lower than that calculated from 1Cx. Moreover, the samples behave differently, according to their degree of ploidy (**Supplementary Figure S5B**). Therefore, we confirm that, as other methods, LocoGSE is indeed a monoploid genome size (1Cx) estimator.

### 2.4 Benchmark (data and programs)

#### 2.4.1 Readsets

In order to benchmark LocoGSE, we downloaded short reads datasets for 8 Angiosperm species broadly distributed across the phylogeny and with genome sizes (1Cx) ranging from 157 Mb to 17.5 Gb and sequencing depths (calculated from Kew 1Cx) ranging from 0.64X to 60.1X (**Supplementary Table S3**). We also downloaded genome assemblies in order to test mapping based approaches, when available (all species except *Papaver nudicaule*). Among the read sets selected, some were also included in the benchmark performed by Pucker et al. for MGSE: *V. vinifera* and *Z. mays* (Pucker, 2019). One sample is highly heterozygous (*Vitis vinifera*) and one is tetraploid (*Solanum tuberosum*). For *Vitis vinifera*, since the readset was very large (380X), we randomly selected 10% of the read pairs to perform our benchmark. For *Solanum tuberosum* and *Zea mays*, we downloaded only one of the two files, containing one read from each pair. For *Allium cepa*, we downloaded two read sets and ran the prediction on each one independently, in order to check the consistency of the results (**Supplementary Table S4**).

#### 2.4.2 Programs

We compared LocoGSE with five short reads-based genome size estimators: three based on k-mers [GenomeScope2.0 (Ranallo-Benavidez et al., 2020), CovEst (Hozza et al., 2015) and RESPECT (Sarmashghi et al., 2021)] and two based on mapping [ModEst (Pfenninger et al., 2022) and MGSE (Pucker, 2019)].

Before running GenomeScope and CovEst, we first generated the k-mer spectrums using Jellyfish (Marçais and Kingsford, 2011), either with k-mers of 21 or 31. The CPU time and memory usage of Jellyfish were included in the performances measured for GenomeScope and CovEst.

GenomeScope (version 2.0) was downloaded at https://github.com/tbenavi1/genomescope2.0. We set up the parameters to k=31 since it is a broadly used k-mer size and used default ploidy (p=2): the output “genome haploid length” corresponds to 1Cx.

CovEst was downloaded at https://github.com/mhozza/covest and launched with k=21 (default) or k=31, and with -m repeats since plant genomes are known to be rich in repeated elements (as expected, tests with the default option (-m basic) produced less accurate genome size estimations).

RESPECT was downloaded from https://github.com/shahab-sarmashghi/RESPECT. It is to be noted that RESPECT requires the installation of an academic licence for Gurobi, and thus can not be run on any computer, which is a limitation. RESPECT was launched with default parameters, on all reads and also on reads filtered with Kraken2 (Wood et al., 2019) to remove human and bacterial contamination according to the authors’ recommendations. We used the standard database downloaded from https://genome-idx.s3.amazonaws.com/kraken/k2_standard_20230314.tar.gz. Kraken unclassified reads (option –unclassified-out) were provided as input for RESPECT. Genome size estimations obtained with or without filtering the reads were very similar, which suggests that the datasets were not very contaminated. The CPU time consumption was lower on the filtered reads, but when adding the time necessary to run Kraken2, the performances were comparable (**Supplementary Table S4**).

Before running mapping based predictors, we first aligned the reads onto the assemblies using BWA-MEM (Li, 2013). MGSE was downloaded from https://github.com/bpucker/MGSE. It was launched using BUSCO annotations, which required first running BUSCO (Simão et al., 2015) on the assembly, with options “–augustus –lineage embryophyta_odb10”. We then provided MGSE with the BUSCO output directory (–busco option).

ModEst was downloaded from https://github.com/schellt/backmap and launched with default parameters. In one case (*Z. mays* readset), ModEst failed and provided a fake depth of “1”, leading to an aberrant size estimation instead of generating an error message. After relaunching several times, the same error occurred.

For each program, we monitored the memory consumption and the CPU time (user+system) (**Supplementary Table S4**). For each genome size prediction, we also calculated the % of error as:


(Predicted Size − Expected Size Kew) x 100/Expected Size Kew

### 2.5 Genome size predictions on read sets at various sequencing depths

In order to evaluate the sequencing depth required for the genome size predictors, we built datasets with various depths of coverage for *Chenopodium suecicum* by randomly selecting read pairs in the fastq files (SRR4425238, total= 28.9X) (**Supplementary Table S5**). Subsampling was performed using an in-house program, getRandomSeq, available at https://github.com/institut-de-genomique/saturn. For each depth, we ran LocoGSE, GenomeScope 2.0 (k=31), RESPECT and CovEst (with k=31 since the prediction was more accurate than for default value of k=21 on this dataset), and computed the average and standard deviation for the predictions as well as the % of error of the predictions, by comparison to the 1Cx reference value obtained from Kew db.

### 2.6 Ploidy estimation

We downloaded 12 read sets from *Senecio doronicum* specimens with various degrees of ploidy (**Supplementary Table S6**) and for which the 1C genome size was estimated by flux cytometry (Fernández et al., 2022). We used LocoGSE to estimate 1Cx genome sizes, and we estimated the ploidy level of each sample with the following formula:


$$Estimated\;ploidy=2\;*\frac{1C\;\left(cytometry\right)}{1Cx\;\left(LocoGSE\right)}$$

## 3 Results

### 3.1 Genome size predictions obtained on 430 Angiosperm species (used for calibration)

**Figure 3:**
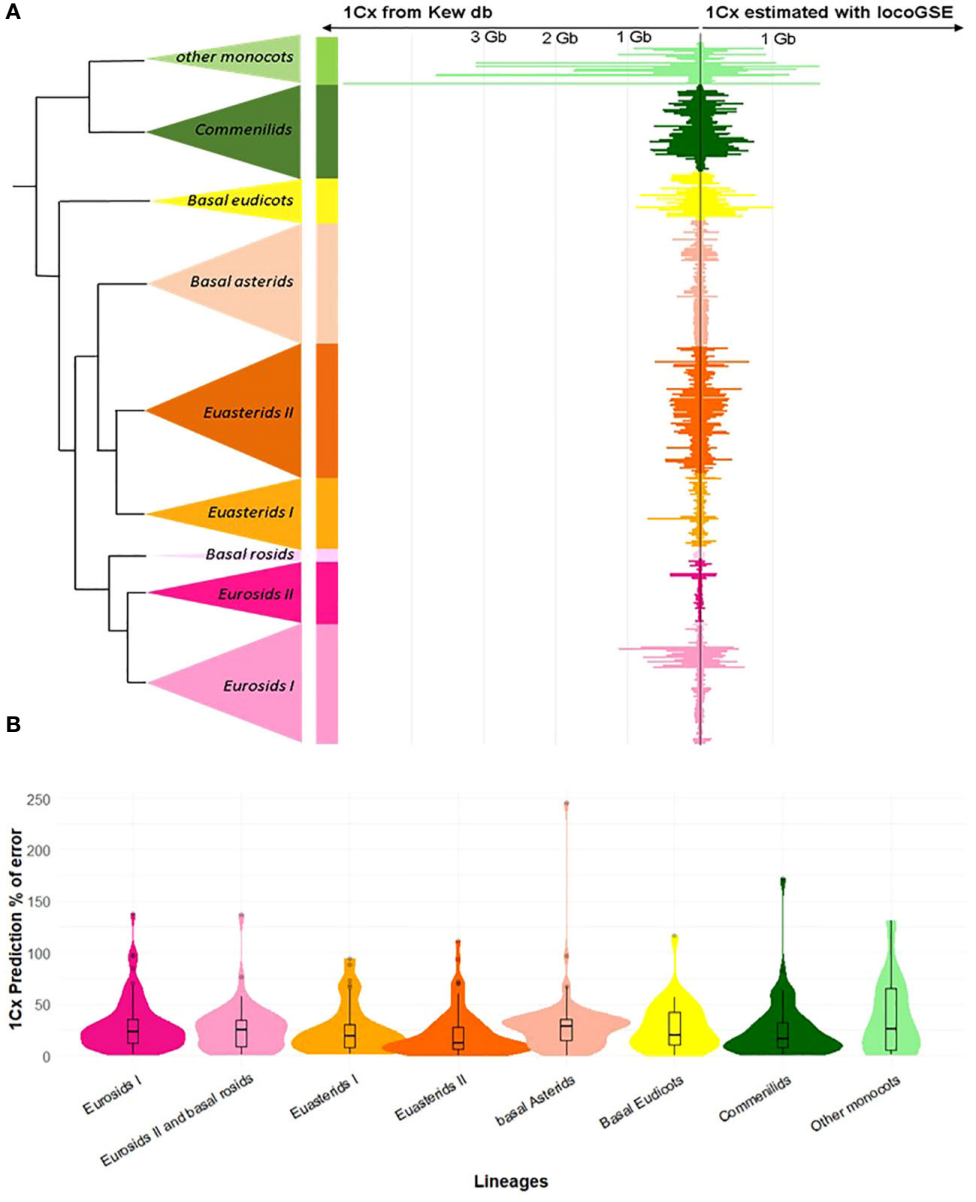
Results of genome size estimation with LocoGSE on the training set. **(A)**. Comparison of expected (left: Kew 1Cx sizes) and predicted sizes (right: LocoGSE output) for the 430 species of the training set, ordered by phylogeny. **(B)**. Violin plots representing the % of error of LocoGSE predictions by plant lineage. Median % of errors for each lineage are displayed above plots.

As a first check for the validity of the approach, we compared genome sizes predicted by LocoGSE with expected 1Cx genome sizes from Kew database on the 430 plant samples used for the calibration of the method (**Figure 3**). For most lineages, the predictions were accurate (mean error rates are between 0.21 for Euasterids II and 0.37 for other monocots). However, sizes were underestimated for very large genomes (in particular “other monocots”: **Supplementary Figure S6**). This observation can be explained by the fact that genome skimming experiments provide a very low depth of coverage (**Supplementary Figure S7**) for such large genomes, and the estimation of mapping depth on single copy proteins with less than 1 read per gene becomes very uncertain. As it is not surprising that the results obtained on the training set used to calibrate the method are accurate, we benchmarked LocoGSE and other genome size estimators on independent datasets.

### 3.2 Comparison of LocoGSE with other genome size estimators

#### 3.2.1 Prediction accuracy

**Figure 4:**
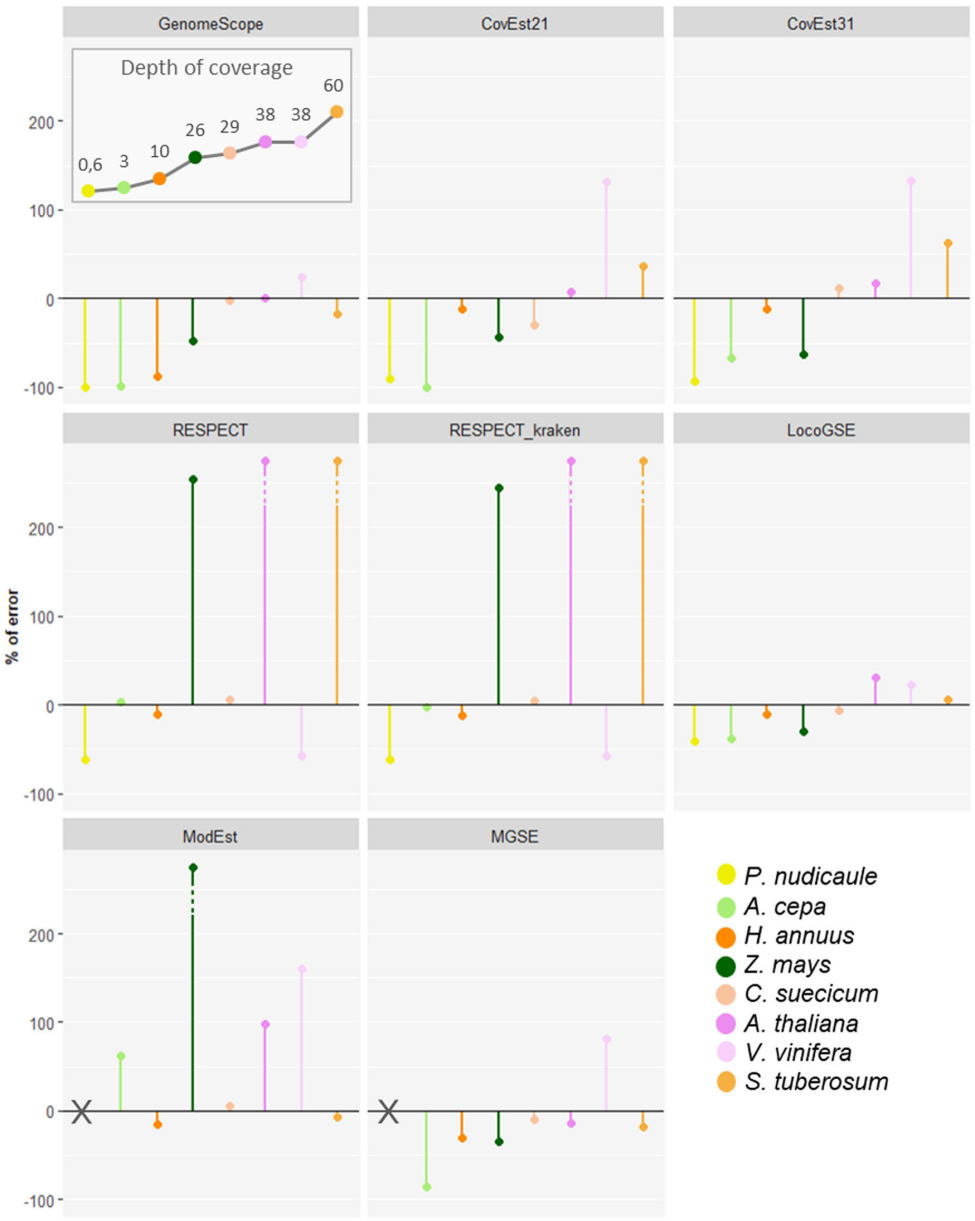
Percent of error (relative to Kew 1Cx) obtained for genome size predictions for 6 genome size estimators on 8 plant sequencing datasets, with various sequencing depths of coverage. Plant species are ordered from left to right from the lowest to the highest sequencing depth as displayed in the “depth of coverage” panel: points are connected for easier visualization.

We downloaded read sets that were not used for the calibration and correspond to eight species from all major plant lineages with various expected sizes and various sequencing depths (**Supplementary Table S3**). We compared LocoGSE predictions with five other genome size prediction softwares (two k-mer based and two assembly mapping-based) (**Supplementary Table S4**, **Supplementary Figures S4**, **S8**). For CovEst, we tested two values of k-mer (the default k=21, and the more commonly used k=31): the results were similar, and the best prediction was alternatively the one with k=21 or k=31. We tested RESPECT with and without removing contaminant reads with Kraken (see Methods): again, results were very similar. For subsequent comparisons, we focused on default options for all programs (**Table 1**). Kew 1Cx estimates were used as the reference for genome size because complete (T2T) assemblies were not available for all plant lineages that were included in the benchmark. Moreover, the added-value of Kew genome sizes is that they are obtained by an orthogonal method (i.e. flow cytometry, that is not sequence-based) compared to the sequence-based genome size estimators that are benchmarked. As a matter of comparison, **Table 1** also displays the size and level of the most complete assembly for each species. At very low depth of coverage (*P. nudicaule* 0.6X and *A. cepa* 3X), LocoGSE and RESPECT were the only softwares to provide non aberrant genome sizes. At low depth (*H. annuus* 10X), all softwares except GenomeScope provided acceptable results, but the best prediction (with regard to Kew estimate) is the one provided by LocoGSE. When considering the chromosome level assembly size as reference, four predictors including LocoGSE provide very accurate predictions (error rate<0.1). Above 25X, all programs are able to make acceptable predictions, but some produce aberrant predictions for some read sets (ModEst for *Z. mays*, which is probably due to the error reported in Methods, RESPECT for *Z. mays*, *A. thaliana* and *S. tuberosum*, which is not surprising since it was designed for low coverage samples) (**Supplementary Figure S8**). For *Vitis vinifera*, which is a highly heterozygous sample, the predictions that were within an acceptable range of the expected size were the ones provided by LocoGSE and GenomeScope. For *Solanum tuberosum*, which is a tetraploid, LocoGSE, GenomeScope and the two mapping-based approaches provided predictions that are close to 1Cx, strongly supporting our observation that such approaches are expected to estimate 1Cx rather than 1C (**Figure 1**). In summary, predictions made by LocoGSE are never aberrant (their %error range is the lowest of all predictors: **Figure 4**), and often the best ones (**Table 1**, **Supplementary Figure S8**). It is to be noted that few genome size predictions are below the threshold of 10% of error, underlining the difficulty of sequence-based genome size estimation.

**Table 1:** Genome size estimations obtained with 6 predictors applied to 8 plant datasets with default parameters. More extensive information can be found in **Supplementary Table S4**. Estimators are ranked according to the error rate (absolute value), calculated as explained in Methods by comparison with 1Cx obtained from Kew db and assembly size (when available). All genome sizes are provided in Megabases.

Species	*Papaver nudicaule*			*Allium cepa*			*Helianthus annuus*			*Zea Mays*			*Chenopodium suecicum*			*Arabidopsis thaliana* col-0			*Vitis vinifera* (Chardonnay)			*Solanum tuberosum*		
Lineage	Basal Eudicots			Other monocots			Euasterids I			Commenilids			Basal Asterids			Eurosids II			Eurosids I			Euasterids II		
SRA_ID	SRR17698145			ERR5262394			SRR5004592			SRR1575500			SRR4425238			SRR1810274			SRR7141304			SRR15198297		
sequencing depth (after trimming to 100nt)	0.65 (0.43)			3.44 (2.73)			9.60 (9.60)			25.61 (25.61)			28.87 (28.87)			37.71 (37.71)			38.03 (25.46)			60.10 (39.80)		
1Cx from Kew (Mb)	4018			17542			3597			2646			739			157			392			948		
Most complete assembly length (Mb)	No assembly			15932^+^			3010^+^			2288^+^			537			142**^++^**			490			775^+^		
GS LocoGSE*	2338	1	-	11836	2	2	3212	1	4**	1859	1	1	688	4**	3	204	4	4	481	1	2**	1003	1**	4
GS GenomeScope 2.0 (k=31)*	10	4	-	205	5	5	425	6	6	1366	4	4	719	1**	4	158	1**	2	488	2	1**	780	3	1**
GS CovEst (k=21)*	355	3	-	10	6	6	3183	3	2**	1480	3	3	515	6	1**	168	2**	3	907	5	5	1289	5	5
GS RESPECT*	1532	2	-	18844	1**	1	3199	2	3**	9384	5	5	781	2**	5	3176	6	6	168	3	4	85665	6	6
GS ModEst*	No assembly	-	-	28050	3	3	3070	4	1**	64500	6	6	783	3**	6	310	5	5	1020	6	6	887	2**	3
GS MGSE (BUSCO)*	No assembly	-	-	2800	4	4	2487	5	5	1728	2	2	671	5**	2	135	3	1**	713	4	3	780	3	1**

^+^:assemly level=chromosome.

^++^:assemly level=complete.

*:2nd col=rank of the estimator with ref=Kew, 3rd col= rank of the estimator with ref=Assembly.

**:less than 10% error (bold).

#### 3.2.2 Performances

**Figure 5:**
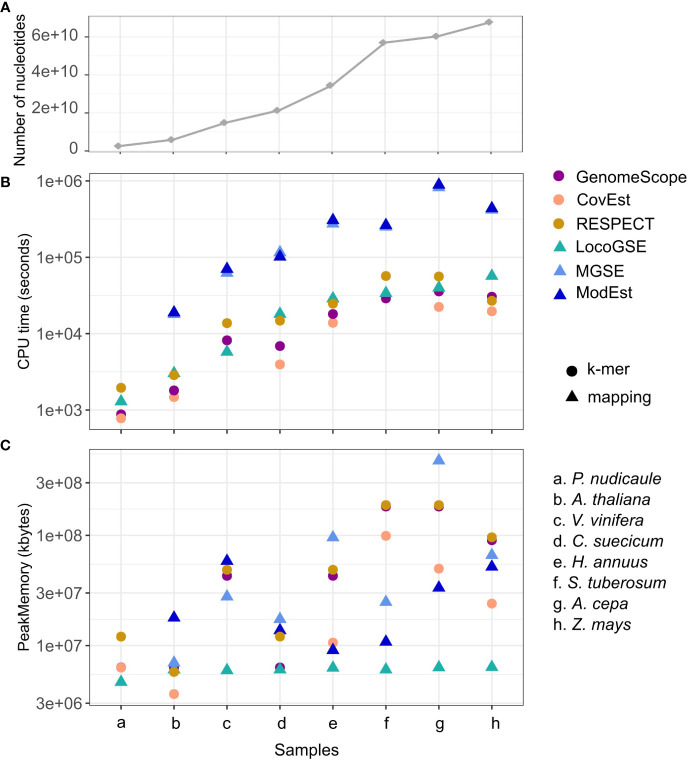
Performances of 6 genome size estimators for 8 plant short read datasets. **(A)** number of nucleotides in the sample. **(B)** CPU time (log scale) and **(C)** Peak Memory. Datasets are ordered from the lowest to the highest number of nucleotides treated (the number of reads and their lengths can be found in **Supplementary Table S3**), and points in panel **(A)** are connected for easier visualisation. K-mer-based methods are represented with circles, and mapping-based methods with triangles.

We compared CPU run time and memory consumption for the six programs with default parameters (**Supplementary Table S4**). **Figure 5** displays the program performances, with the datasets (species on the x axis) ordered from the lowest to the highest number of nucleotides in the inputted readsets. As expected, for all programs, the running time increases when increasing the number of nucleotides (**Figure 5A**). Additionally, the CPU time is one order of magnitude higher for the two assembly mapping-based methods (this is caused by the step of mapping the reads on the assembly). LocoGSE requires only to map the reads on a few hundred of single copy proteins, which explains why it is faster. As expected, k-mer based approaches are the least time-consuming. However the algorithm used in RESPECT relies on a complex modeling of genomic parameters (Sarmashghi et al., 2021) and thus performs slower. In summary, the CPU time for LocoGSE is comparable to that of RESPECT and intermediary between k-mer and assembly mapping-based methods. As CPU time, memory usage also increases with the number of nucleotides treated but LocoGSE is a notable exception: memory usage remains constant and low for all runs (**Figure 5B**).

#### 3.2.3 Required sequencing depth

**Figure 6:**
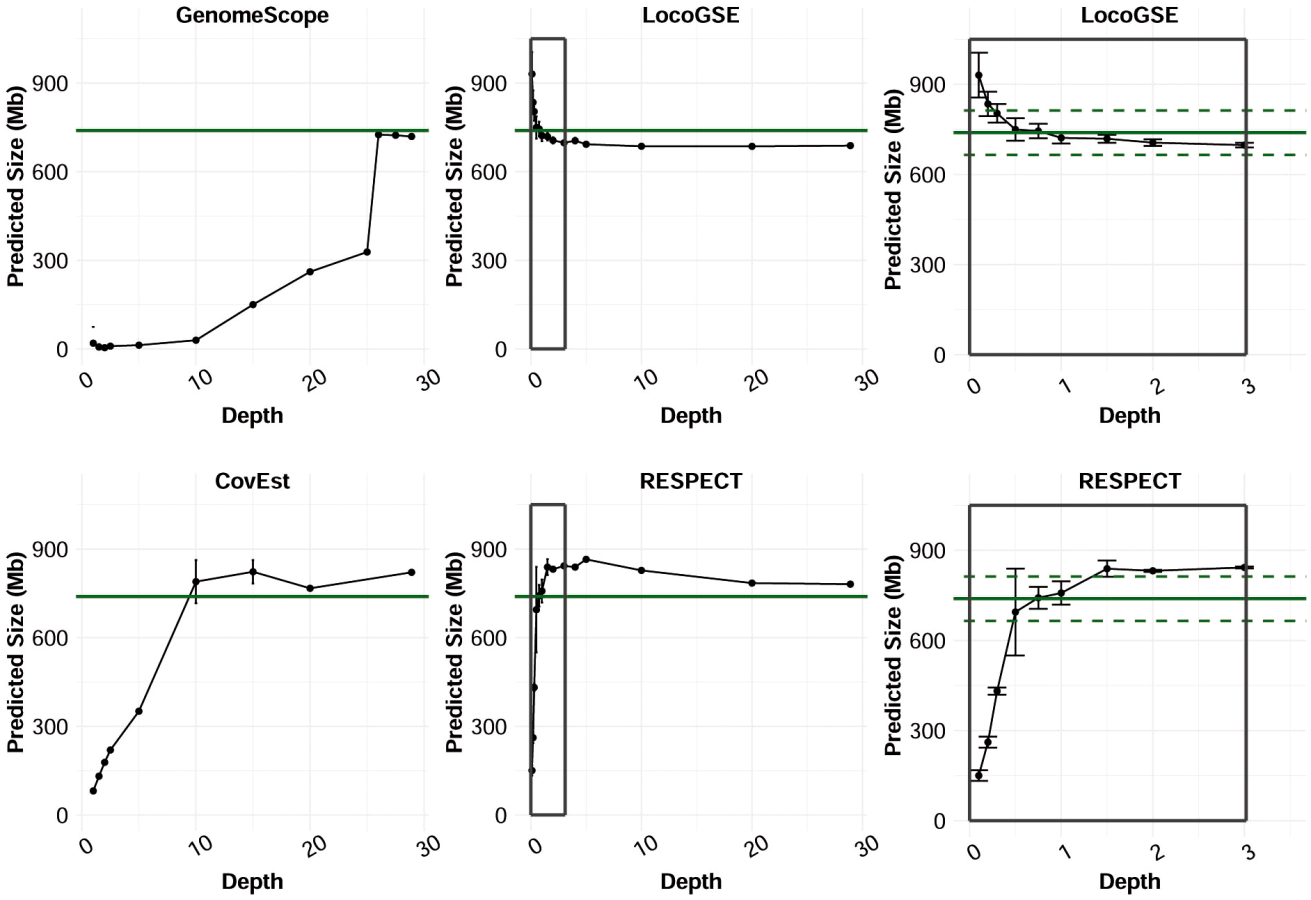
Genome size estimations made by predictors on subsets of reads of *C. suecicum* with increasing depth of coverage, from 0 to ~30X, and zoom on 0 to 3X for LocoGSE and RESPECT. Several runs (up to 10) were performed for each depth: dots are positioned at the average prediction and error bars represent one standard deviation up and one standard deviation down. Green continuous line shows the expected genome size (Kew 1Cx estimate: 739 Mb) and dashed lines the 10% error interval.

In order to identify the short read sequencing depth required for each software to provide accurate predictions, we compared the genome size estimations obtained from read sets sampled from the same sequencing run at various depths (**Figure 6**). We focused on the three k-mer based programs and LocoGSE, since the need for an assembly precludes the use of MGSE and ModEst on low coverage datasets. We selected the readset from *Chenopodium suecicum*, because all estimators provided accurate genome size predictions when using the whole dataset (28.9X) (**Supplementary Figure S8**). First, it is notable that predictions are usually very consistent between samples (**Supplementary Table S5**). Nonetheless, as expected, LocoGSE predictions are more variable between samples (larger error bars) at extremely low depth of coverage (<0.5X) than at higher depths. Moreover, all predictions appear to reach a plateau after a certain depth. Interestingly, the plateau is reached at low depths for LocoGSE and RESPECT, whereas it is reached at 10X for CovEst, and 26X for GenomeScope (**Figure 6A**). When comparing the two packages designed for very low coverage datasets, we notice that both LocoGSE and RESPECT reach a plateau at very low depths (0.5X). Notably, LocoGSE provides better predictions at extremely low depth (0.1X) and converges faster than RESPECT towards accurate estimations (**Figures 6B, C**). Both tools are very accurate on the complete *C. suecicum* dataset but it remains to be noted that RESPECT is not recommended for high coverage datasets (Sarmashghi et al., 2021), and was shown to produce erroneous estimations on other read sets at high coverage. In particular, for the *Arabidopsis thaliana* dataset, with a depth of coverage of 38X, RESPECT estimated a genome size of 3,176 Mb instead of the expected 157 Mb (**Table 1**).

In conclusion, LocoGSE is the only software that can be used at any sequencing depth, and although the running time is higher for LocoGSE compared to k-mer based approaches like GenomeScope for a given readset, the number of reads required is much lower (1X vs >25X). Consequently, the running time needed to get a reasonable prediction is actually lower for LocoGSE than GenomeScope.

### 3.3 Comparison of predictions for various degrees of ploidy

**Figure 7:**
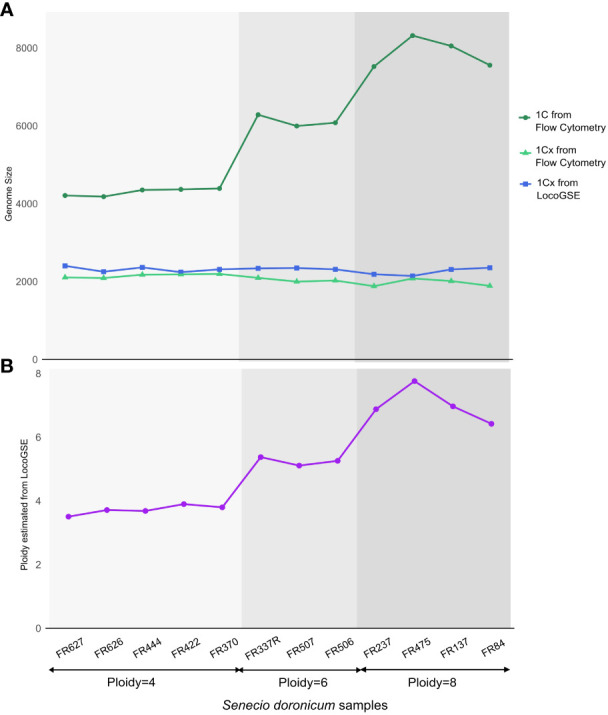
Genome size and ploidy estimations for 12 *Senecio doronicum* specimens with various ploidy levels (4, 6, 8). **(A)** genome size estimated by flow cytometry (blue) and LocoGSE (green). **(B)** ploidy estimated by dividing Kew 1C by LocoGSE 1Cx (purple). Samples are ordered according to their ploidy levels and points are connected for easier visualization.

We wanted to investigate the sensitivity of the method to the ploidy level of the input samples. For that purpose, we used various read sets from *Senecio doronicum* specimens, with ploidy levels ranging from 4 to 8 (Fernández et al., 2022) (**Supplementary Table S6**). As seen in **Figure 7**, 1Cx genome sizes estimated with LocoGSE are close to expectations and stable across all samples, regardless of the ploidy level. We can thus conclude that LocoGSE is effectively estimating 1Cx rather than 1C, as expected from our model (**Figure 1**). We estimated the ploidy level using 1C genome size measured by flow cytometry and 1Cx genome size estimated by LocoGSE. Estimated ploidies are close to observed ones. Notably, for higher degrees of ploidy, the ploidy levels tend to be slightly underestimated as a consequence of lower 1C estimation by flow cytometry but constant 1Cx estimation by LocoGSE. This result could be attributed to genome downsizing after polyploidy, that probably did not affect single copy OneKP genes as much as the rest of the genome, thus resulting in a slight overestimation of the 1Cx genome size by LocoGSE.

## 4 Discussion

We developed LocoGSE, a monoploid genome size estimator aimed at using short reads at very low coverage. Although it is not often stated in the documentation of other genome size estimators, all sequence-based genome size estimators (k-mer or mapping based) predict 1Cx rather than 1C, since they are not able to distinguish k-mers or reads from different haplotypes under a certain threshold of heterozygosity. GenomeScope 2.0 (Ranallo-Benavidez et al., 2020), the most widely used k-mer based method, is able to cope with highly heterozygous and polyploid samples. The so-called “genome haploid length” it predicts indeed corresponds to 1Cx. But it is to be noted that GenomeScope needs the user to provide a ploidy value in order to estimate 1Cx accurately. Otherwise, it is estimated with the default value of 2. Since most lineages are diploids, the absence of ploidy information is not often problematic: the estimated 1Cx value is equal to 1C in this case. We believe this is the reason why “genome size estimators” do not document the fact that the estimation they provide corresponds to 1Cx (monoploid genome size). But for lineages where polyploidy is frequent, like plants (Heslop-Harrison et al., 2023), genome size predictors will need to be combined with short-reads based reference-free ploidy estimators such as SMUDGEPLOT (Ranallo-Benavidez et al., 2020) or PLOIDYFROST (Sun et al., 2023) in order to obtain an estimation of 1C genome size. In this aim, there is a need to develop ploidy predictors that are able to cope with low coverage datasets. Nevertheless, monoploid genome size (1Cx) predictions also have inherent value: they can be used to estimate the ploidy level of a specimen, provided flow cytometry is performed to estimate 1C. Such an approach could be easier to implement than classical microscopy approaches to infer the number of chromosomes and be of great use for botanists.

LocoGSE relies on deducing the sequencing depth from the depth of mapping of short reads on single copy genes. The fact that the coefficient (slope) linking depth of mapping on the single copy genes and sequencing depth is not equal to 1 could seem counter-intuitive. In the model described by Pflug et al., the coefficient is assumed to be 1, which is expected when mapping the reads on complete gene sequences, at the DNA level (Pflug et al., 2020). Contrastingly, for LocoGSE, translated short DNA reads are mapped on protein sequences. Consequently, various possible biases are likely to impact the quality of mapping (and subsequent depth calculation) and explain why different coefficients are found for different phylogenetic branches. First, the phylogenetic distance between the sample studied and the consensus for monocopy genes will have an impact on the number of reads mapped. However, we showed that the OneKP consensus, unlike BUSCO, aligns with similar percent identities on all Angiosperm lineages (**Supplementary Figure S3**), suggesting it is evenly distant to all lineages, which is the reason why we selected it as the default dataset. Additionally, for lineages with different numbers of exons/introns per gene, the results of the mapping will be different, since reads will not map on exon-exon junctions. Since OneKP genes are ancestral to all *Viridiplantae*, intron/exon structures of these genes are expected to be relatively stable, but in the event of species harboring very small exons (shorter than 100nt, the length or trimmed reads mapped), we expect LocoGSE not to be able to predict genome size accurately. Also, for polyploids that are in the process of diploidization by loss of copies of genes to go back to the diploid state (Langham et al., 2004), the signal can be noisy because some of the supposed “single copy” genes are in several copies and others in single copy, leading to a predicted genome size between 1Cx and 1C. Finally, LocoGSE is expected to be sensitive to contaminations in the read sets (bacterial DNA, chloroplastic DNA), because it is relying on the Lander-Waterman equation, and even more so when it is used on low coverage readsets. For this reason, we filtered out two genome skimming read sets from the training set (used for calibration), because they contained more than 20% of bacterial reads (all others had less than 6% of bacterial reads). We used Kraken2 on the read sets used for benchmarking the 6 genome size estimators and showed that at most 7% of the reads correspond to bacterial or human contamination. Such low percentages allow accurate gene prediction, but one should keep in mind that it is recommended to check the level of contamination of the read sets before running any sequence-based genome size estimator.

As already mentioned, LocoGSE requires a calibration step using short reads datasets corresponding to genomes with known 1Cx sizes. This was achieved using a large dataset of plant genome skims as well as carefully curated genome size prime estimates from Kew Plant DNA C-values database. While flow cytometry may not always be entirely accurate, Kew size estimates stand out as the closest approximation to the ground truth that we have at our disposal for a very large number of species. Moreover, we carefully selected the species to include in the calibration set among the ones with low 1Cx variation among cytotypes, in order to overcome the effect of intraspecies variation of monoploid genome size. Indeed, intraspecies genome size variation has been described in various plants (Schmuths et al., 2004; Díez et al., 2013; Long et al., 2013; Bilinski et al., 2018; Becher et al., 2021; Balant et al., 2022) and genome size should be considered as a trait of individuals rather than species. Consequently, users should be aware that size estimations generated by any method correspond to the analyzed individual and are not necessarily representative of the whole species.

Using LocoGSE on plants is very straightforward, because of the added-value provided by the calibration we performed. For other lineages, the user will need to input a set of single copy genes corresponding to the organism studied. BUSCO database provides gene sets for a wide range of species (Simão et al., 2015; Manni et al., 2021). Alternatively, one can build orthogroups for a specific lineage, detect single copy gene families, and generate consensus sequences for these families, as was done recently to develop a single copy genes resource for coral genomes (Noel et al., 2023). Then, if possible and instead of using a default slope of 1, the user can calibrate LocoGSE using sequencing data from genomes of known sizes, as explained in our wiki (https://github.com/institut-de-genomique/LocoGSE/wiki) (**Supplementary Figure S2B**).

To summarize, we showed that LocoGSE fills a gap in sequence-based genome size estimation for several reasons. First, the depth of coverage required to get optimal predictions is as low as 0.5X, and no reference assembly is required, which makes LocoGSE particularly suitable for genome skimming datasets. LocoGSE can also find its use in biodiversity sequencing projects in which an inexpensive and superficial first sequencing experiment can provide a lot of information including genome size. Another package, RESPECT (Sarmashghi et al., 2021), also performs very well in the same range of depth. However, its use is complicated by the need to install an academic licence (many research institutions, although public and non-profit, do not have the “academic” status). More worryingly, RESPECT sometimes provides aberrant results when used on higher coverage readsets, which precludes its use on unknown genomes with no previously reported genome sizes. On the contrary, although LocoGSE was calibrated on very low depth of coverage datasets (<2X) and is thus expected to work better for low depths, we showed that the estimations made at higher depths are also accurate. At high coverages (above 30X), users may choose to use k-mer based approaches like GenomeScope, that are less time consuming and were shown to provide accurate results. However, another possible option would be to apply LocoGSE on a subset of reads, which would be more memory-efficient and probably even faster.

Second, as LocoGSE relies on mapping on consensus (ancestral) sequences of single copy genes, it is not sensitive to possible heterozygosity of the considered specimen. Here again, no prior knowledge about the level of heterozygosity or the ploidy of the sample treated is required. Consequently, since LocoGSE can be launched on low coverage sequencing data without any prior knowledge about the properties of the genome considered (size, ploidy, heterozygosity), it can be of great use to inform on sequencing strategies for environmental samples.

Finally, thanks to the calibration already performed, the current version of LocoGSE is readily applicable to most Angiosperm lineages (pre-computed slopes for OneKP (default) and BUSCO Embryophyta single copy gene sets). We will update the dataset with more plants and extend the calibration to Gymnosperms and other vascular plants in the near future.

In conclusion, we believe that LocoGSE will be of great use for the community of researchers in the fields of environmental genomics and plant genome size analysis.

## 5 Collaborators

### 5.1 Members of the PhyloNorway consortium

I.G. Alsos, M.K. Føreid Merkel, Y. Lammers (The Arctic University Museum of Norway, Tromsø, NO), E. Coissac, C. Pouchon (Laboratoire d’Ecologie Alpine, CNRS, UGA, Grenoble, FR); A. Alberti, F. Denoeud, P. Wincker (Génomique Métabolique, Genoscope, Institut François Jacob, CEA, CNRS, Univ Evry, Université Paris- Saclay, FR).

### 5.2 Members of the PhyloAlps consortium

S. Lavergne, C. Pouchon, E. Coissac, C. Roquet, J. Smyčka, M. Boleda, W. Thuiller, L. Gielly, P. Taberlet, D. Rioux, F. Boyer, A. Hombiat, B. Bzeznik (Laboratoire d’Ecologie Alpine, CNRS, UGA, Grenoble, FR); A. Alberti, F. Denoeud, P. Wincker, C. Orvain (Génomique Métabolique, Genoscope, Institut François Jacob, CEA, CNRS, Univ. Evry, Univ. Paris-Saclay, FR); C. Perrier, R. Douzet, M. Rome, J.G. Valay, S. Aubert (Jardin Alpin du Lautaret, CNRS, UGA, Grenoble, FR); N. Zimmermann, R. O. Wüest, S. Latzin, S. Wipf (Swiss Federal Research Institute WSL, Birmensdorf, CH); J. Van Es, L. Garraud, J.C. Villaret, S. Abdulhak, V. Bonnet, S. Huc, N. Fort, T. Legland, T. Sanz, G. Pache, A. Mikolajczak (Conservatoire Botanique National Alpin, Gap, FR); V. Noble, H. Michaud, B. Offerhaus, M. Pires, Y. Morvant (Conservatoire Botanique National Méditerranéen, Hyères, FR); C. Dentant, P. Salomez, R. Bonet (Parc National des Ecrins, Gap, FR); T. Delahaye (Parc National de la Vanoise, Chambery, FR); M.F. Leccia, M. Perfus (Parc National du Mercantour, Nice, FR); S. Eggenberg, A. Möhl (Info-Flora, Bern, CH); B. Hurdu, M. Pușcaș (Babeş Bolyai University, Institute of Biological Research, Cluj Napoca, RO), M. Slovák (Institute of Botany, Bratislava, SK).

## Data availability statement

The original contributions presented in the study are included in the article/**Supplementary Materials**. Further inquiries can be directed to the corresponding author. The program is available at https://github.com/institut-de-genomique/LocoGSE.

## Author contributions

PG-T: Writing – review & editing, Writing – original draft, Software, Methodology. BI: Writing – review & editing, Software, Methodology. IA: Writing – review & editing, Resources. EC: Writing – review & editing, Resources. SL: Writing – review & editing, Resources. J-MA: Writing – review & editing, Validation, Supervision. FD: Writing – review & editing, Writing – original draft, Validation, Supervision, Methodology, Investigation, Conceptualization.
