# Corrigendum: LocoGSE, a sequence-based genome size estimator for plants

**DOI:** 10.3389/fpls.2024.1426035

**Published:** 2024-06-05

**Authors:** Pierre Guenzi-Tiberi, Benjamin Istace, Inger Greve Alsos, The PhyloNorway Consortium, Eric Coissac, Sébastien Lavergne, The PhyloAlps Consortium, Jean-Marc Aury, France Denoeud

**Affiliations:** ^1^Génomique Métabolique, Genoscope, Institut François Jacob, CEA, CNRS, Univ Evry, Université Paris-Saclay, Evry, France; ^2^The Arctic University Museum of Norway, UiT The Arctic University of Norway, Tromsø, Norway; ^3^Univ. Grenoble Alpes, Univ. Savoie Mont Blanc, CNRS, LECA (Laboratoire d’Ecologie Alpine), Grenoble, France

**Keywords:** genome size estimation, genome size, ploidy, genome-skimming, environmental DNA, plant genomics, 1C, 1Cx

In the published article, there was an error in **Figure 2** as published. The same formula “y=1.59x R2 = 0.99” was displayed on all panels, whereas it was supposed to vary across lineages. The corrected **Figure 2** and its caption appear below.

**Figure 2:**
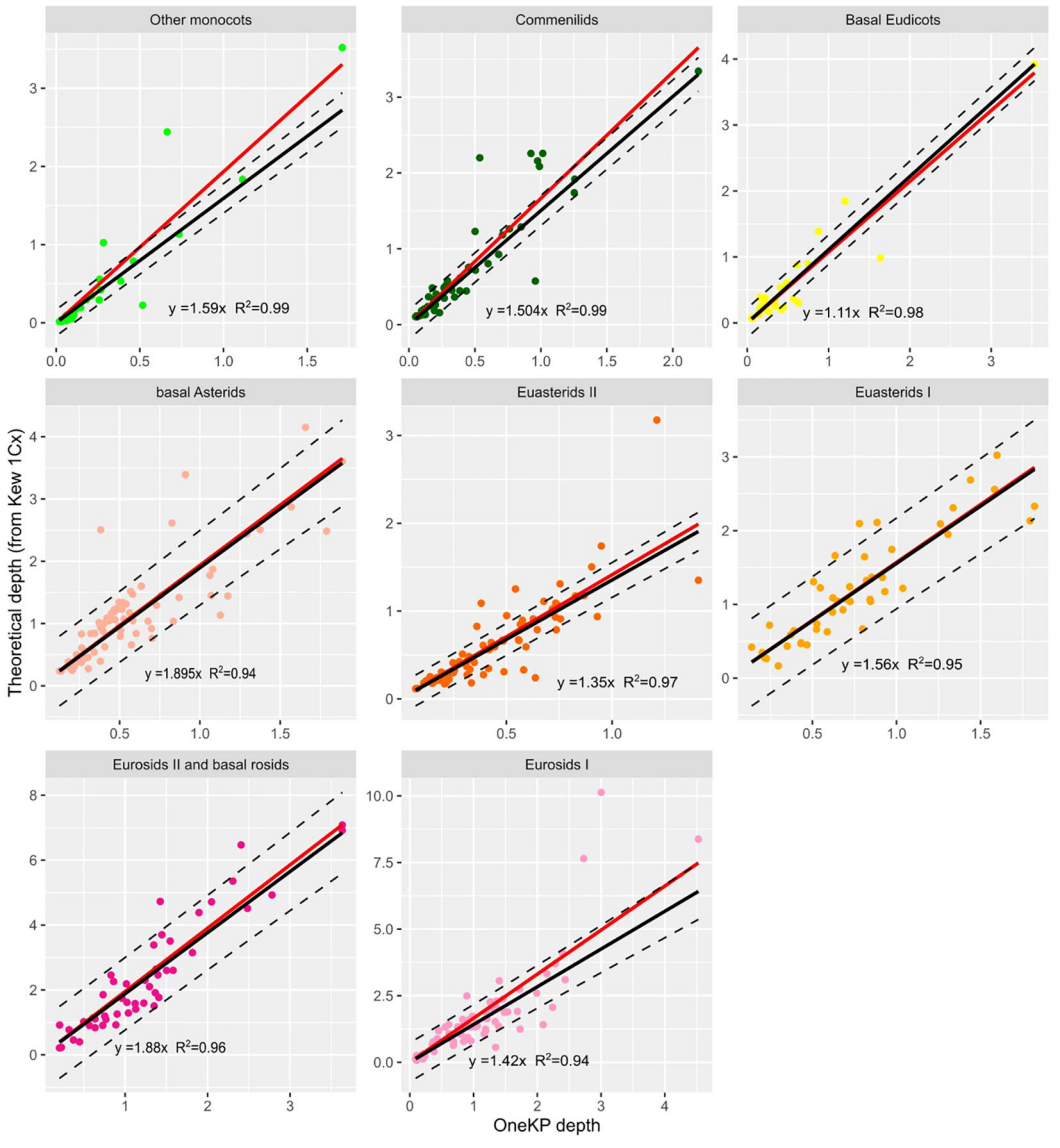
Relationship between depth on OneKP single copy genes and theoretical depth (calculated from Kew 1Cx) in the training set, for 8 plant lineages. Black line is the regression line obtained after robust regression, red line is the regression line obtained with standard regression. Regression line equations and R coefficients are displayed for each lineage.

The authors apologize for this error and state that this does not change the scientific conclusions of the article in any way. The original article has been updated.

