# Supplementary material for: LocoGSE, a sequence-based genome size estimator for plants
Source: Front Plant Sci. 2024 Mar 14;15:1328966. doi: 10.3389/fpls.2024.1328966 (PMC10972871; doi:10.3389/fpls.2024.1328966)

Figure S1. Details of the LocoGSE program

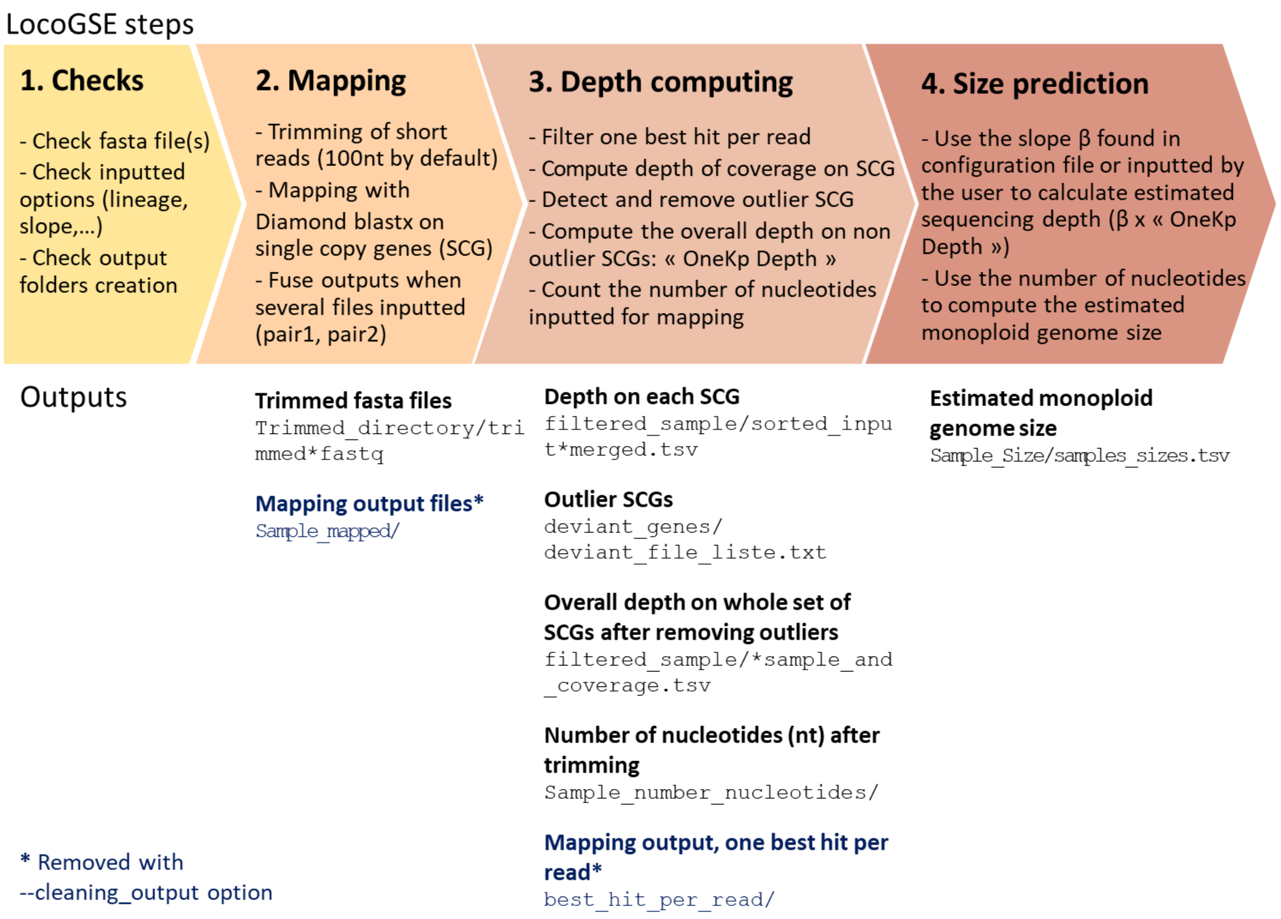

Figure S2 : usage cases of LocoGSE. **A.** For already calibrated species (Angiosperm). **B.** For other lineages, implying a calibration step with known 1Cx values

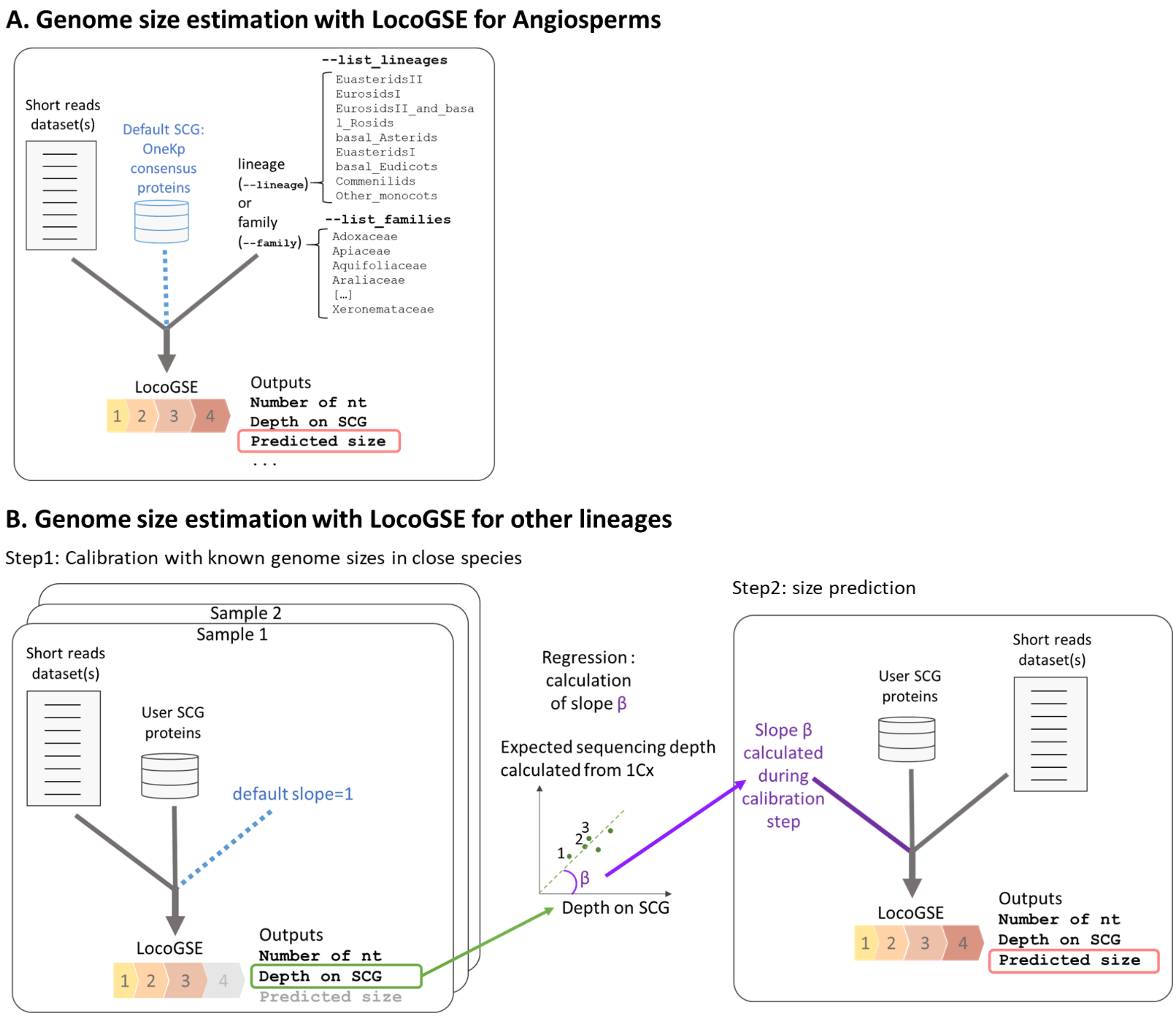

Figure S3. A. Distribution of major Angiosperm lineages in BUSCO Embryophyta (ODB10) and OneKP single copy gene sets, as well as in the dataset used for calibration of LocoGSE. **B.** Boxplots showing the distribution of %identity (at the proteic level: translated reads mapped on proteic sequences) when mapping the reads from the calibration set on Busco (red) and OneKP (green) single copy gene sets, for each phylogenetic lineage.

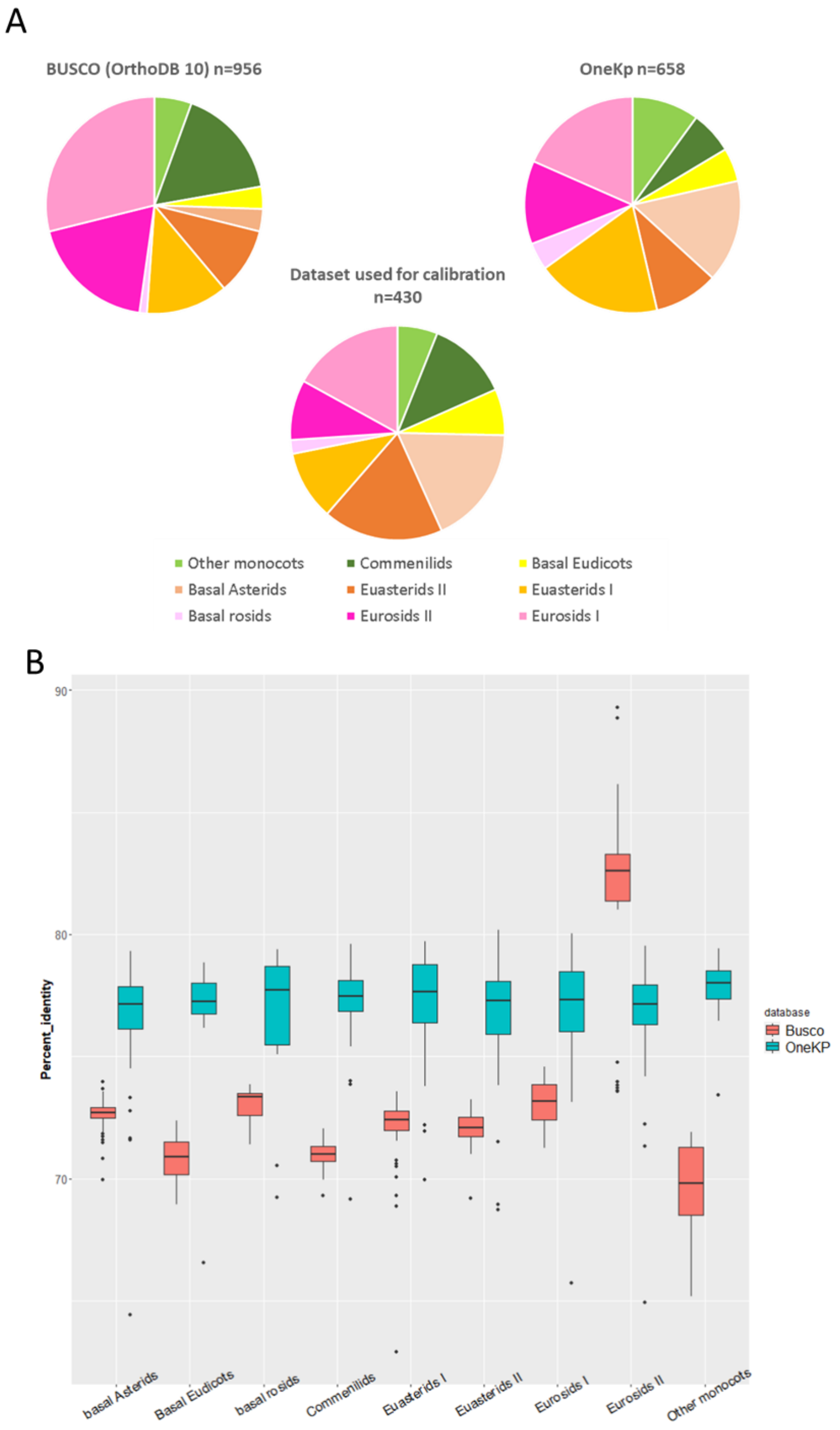

Figure S4. Distribution of the depth of coverage for genome skimming readsets in the training set (caculated from Kew 1Cx).

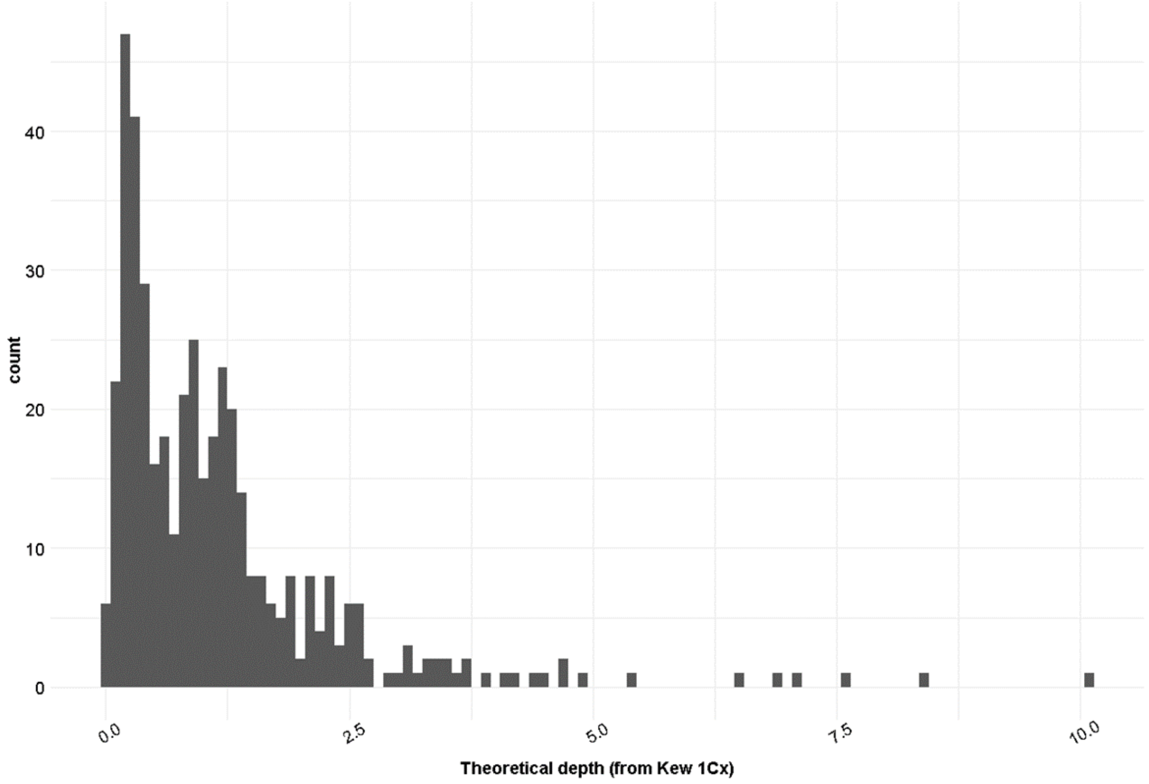

Figure S5. A. Relationship between theoretical 1Cx depth (calculated from Kew 1Cx) and depth on OneKp set for all readsets in the training set. Each dot represents one readset, colors correspond to phylogenetic lineages. **B.** Relationship between theoretical 1C depth (calculated from Kew 1C) and OneKp depth for all readsets in the training set with ploidies of 2, 4 or 8, colored by ploidy level : as expected (Figure 1), slopes decrease with ploidy level.

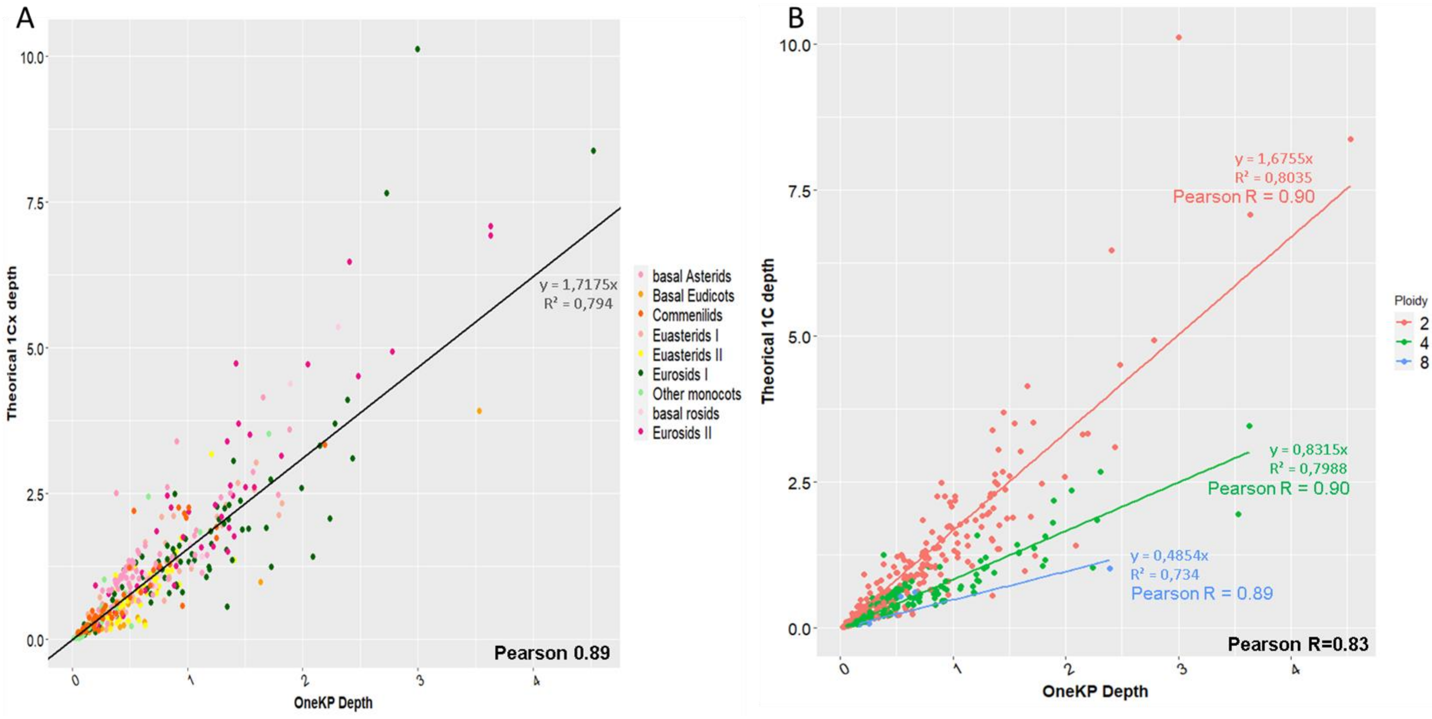

Figure S6. Comparison of 1Cx genome size estimated by LocoGSE (y-axis) with expected Kew 1Cx (x axis), for 9 phylogenetic lineages in the training set.

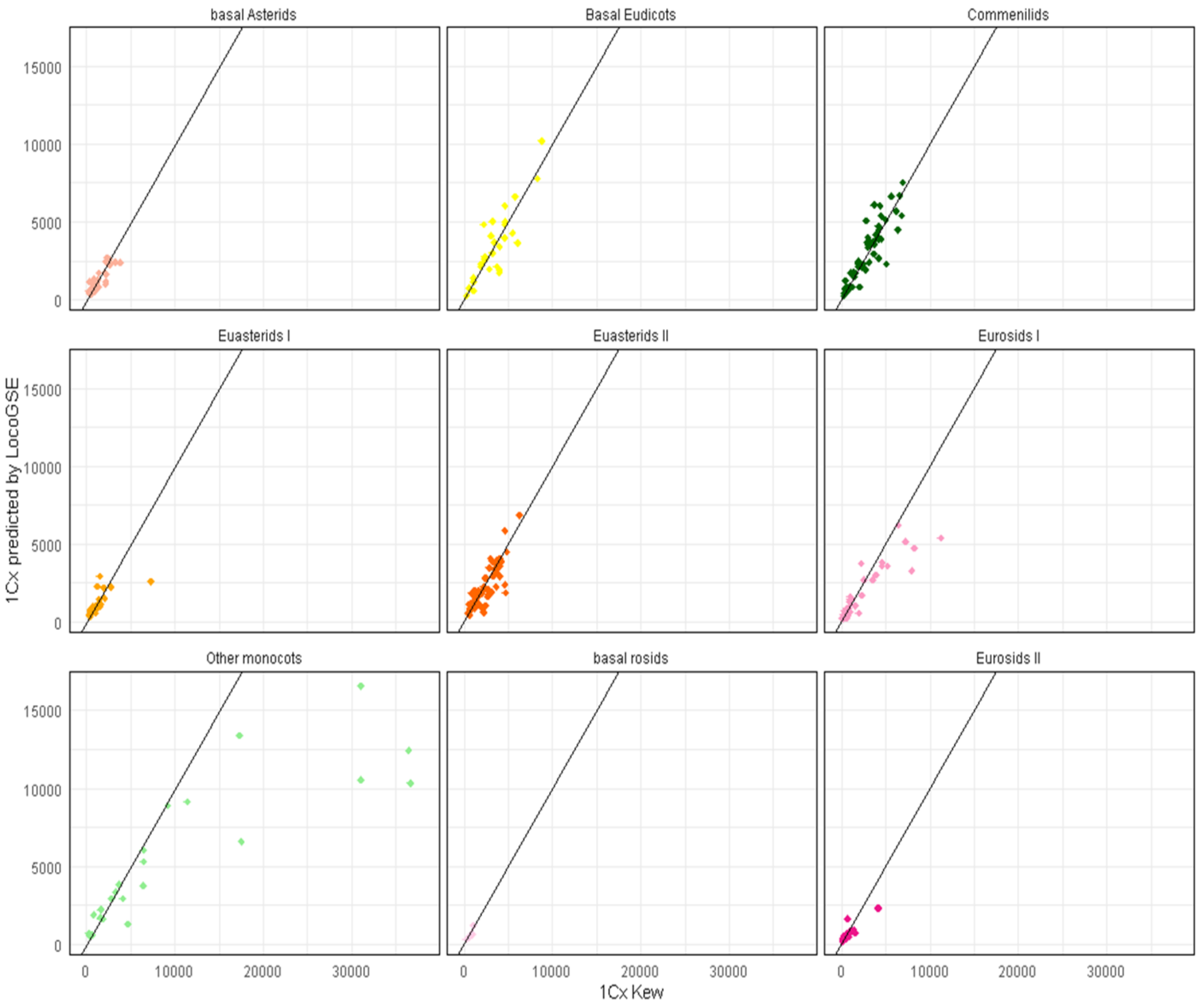

Figure S7. Boxplots showing the distribution of depth of coverage for readsets in the training set, for each phylogenetic branch.

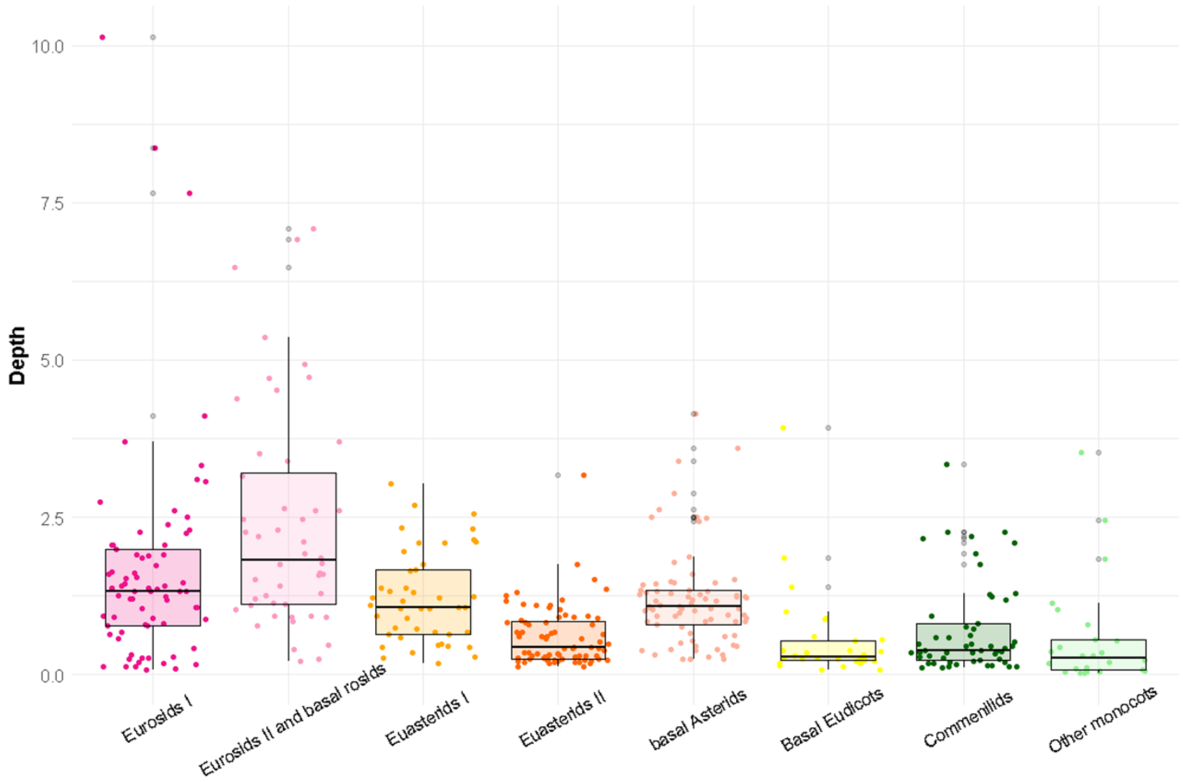

Figure S8. Genome size estimations obtained with 6 predictors on 8 plant readsets. Green dotted lines correspond to expected 1Cx size inferred from Kew database. Predictors are ordered from left to right from the one with the lowest to the highest error rate (absolute value).

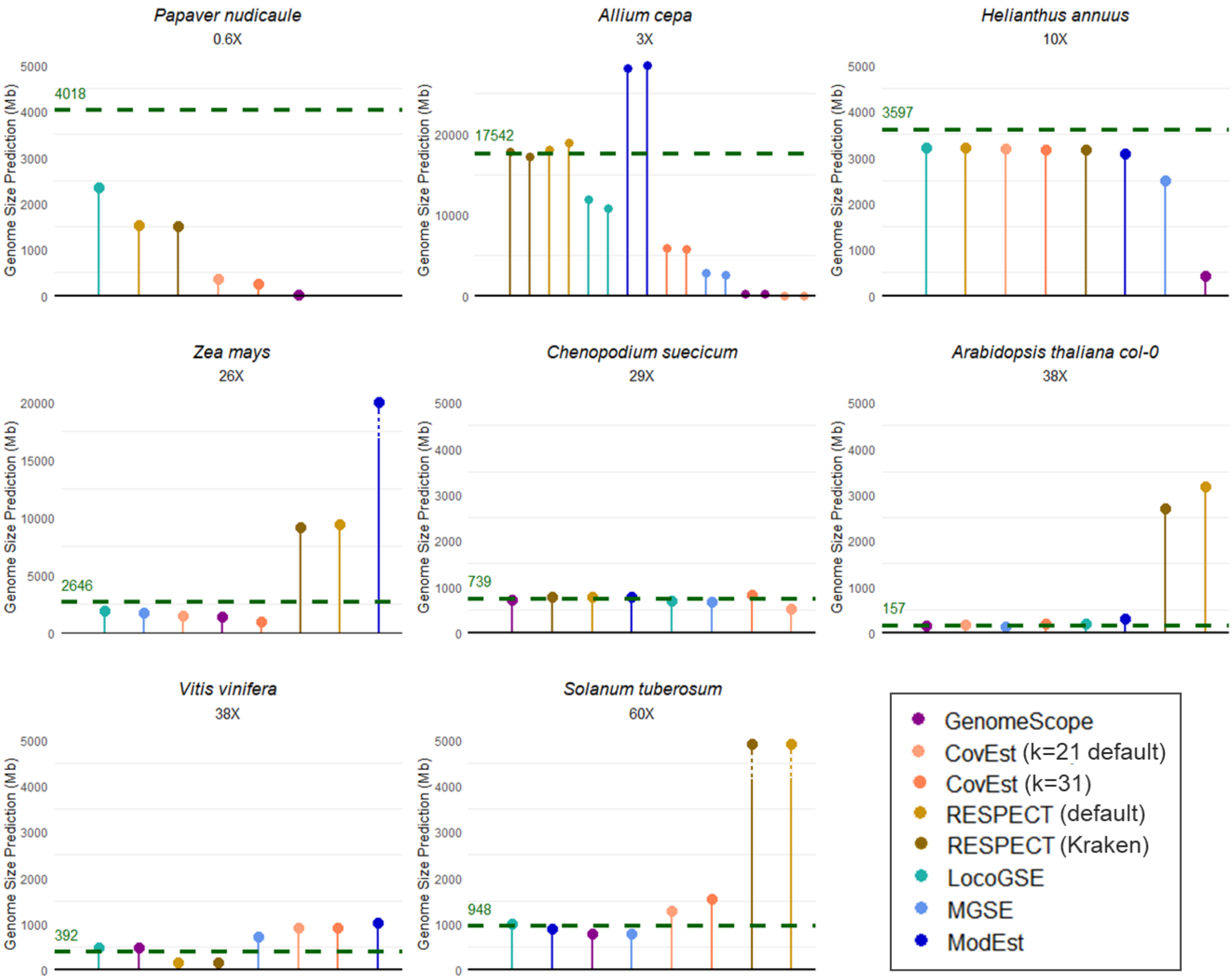

Supplement: Supplementary file 1 [file DataSheet_1.zip › supplementary_figures.pdf]
